# A Comparison Between Chemo-Radiotherapy Combined With Immunotherapy and Chemo-Radiotherapy Alone for the Treatment of Newly Diagnosed Glioblastoma: A Systematic Review and Meta-Analysis

**DOI:** 10.3389/fonc.2021.662302

**Published:** 2021-05-11

**Authors:** Montserrat Lara-Velazquez, Jack M. Shireman, Eric J. Lehrer, Kelsey M. Bowman, Henry Ruiz-Garcia, Mitchell J. Paukner, Richard J. Chappell, Mahua Dey

**Affiliations:** ^1^ Department of Neurosurgery, University of Wisconsin School of Medicine & Public Health, UW Carbone Cancer Center, Madison, WI, United States; ^2^ Department of Radiation Oncology, Icahn School of Medicine at Mount Sinai, New York, NY, United States; ^3^ Department of Neurosurgery and Radiation Oncology, Mayo Clinic, Jacksonville, FL, United States; ^4^ Department of Statistics, Biostatistics and Medical Informatics, University of Wisconsin School of Medicine & Public Health, UW Carbone Cancer Center, Madison, WI, United States

**Keywords:** newly diagnosed glioblastoma, immunotherapy, vaccine, chemo-radiotherapy, high-grade glioma, glioma

## Abstract

**Background:**

Immunotherapy for GBM is an emerging field which is increasingly being investigated in combination with standard of care treatment options with variable reported success rates.

**Objective:**

To perform a systematic review of the available data to evaluate the safety and efficacy of combining immunotherapy with standard of care chemo-radiotherapy following surgical resection for the treatment of newly diagnosed GBM.

**Methods:**

A literature search was performed for published clinical trials evaluating immunotherapy for GBM from January 1, 2000, to October 1, 2020, in PubMed and Cochrane using PICOS/PRISMA/MOOSE guidelines. Only clinical trials with two arms (combined therapy *vs.* control therapy) were included. Outcomes were then pooled using weighted random effects model for meta-analysis and compared using the Wald-type test. Primary outcomes included 1-year overall survival (OS) and progression-free survival (PFS), secondary outcomes included severe adverse events (SAE) grade 3 or higher.

**Results:**

Nine randomized phase II and/or III clinical trials were included in the analysis, totaling 1,239 patients. The meta-analysis revealed no statistically significant differences in group’s 1-year OS [80.6% (95% CI: 68.6%–90.2%) *vs.* 72.6% (95% CI: 65.7%–78.9%), p = 0.15] or in 1-year PFS [37% (95% CI: 26.4%–48.2%) *vs.* 30.4% (95% CI: 25.4%–35.6%) p = 0.17] when the immunotherapy in combination with the standard of care group (combined therapy) was compared to the standard of care group alone (control). Severe adverse events grade 3 to 5 were more common in the immunotherapy and standard of care group than in the standard of care group (47.3%, 95% CI: 20.8–74.6%, *vs* 43.8%, 95% CI: 8.7–83.1, p = 0.81), but this effect also failed to reach statistical significance.

**Conclusion:**

Our results suggests that immunotherapy can be safely combined with standard of care chemo-radiotherapy without significant increase in grade 3 to 5 SAE; however, there is no statistically significant increase in overall survival or progression free survival with the combination therapy.

## Introduction

Glioblastoma (GBM) is the most common primary and dismal brain cancer in adults, this carries a poor prognosis and median overall survival (OS) (1). It is a highly aggressive and heterogeneous entity that survives even the most eradicative treatments (2–4). Current standard of care for GBM includes safe maximal tumor resection, followed by temozolomide (TMZ) chemotherapy (75 mg/m2/day for 6 weeks) and concomitant radiation (60 Gy in 30 fractions). TMZ is then followed by six continued maintenance cycles (150–200 mg/m2/day for the first 5 days of a 28-day cycle); accompanied by the antimitotic device tumor treating fields (TTF) (Optune, Novocure Inc) (4–6), which is continued once TMZ is completed. This standard of care with TTF included, achieves a median overall survival of 20.9 months, that is in contrast with the 16 months median survival obtained with surgery and chemo-radiotherapy alone (7). However, tumor recurrence happens in the majority of the patients despite the aggressive treatment regimen (8), highlighting a major treatment gap in GBM that has yet to be addressed (9–14). Multiple strategies are being developed with the goal of effectively treating GBM, however, one such strategy that has proven viable in other cancer domains and is currently being heavily investigated is immunotherapy (6, 15).

Immunotherapy, is an evolving field of medicine that enhances the activity of select cells in the immune system to recognize, attack, and kill cancer cells *via* targeted anti-tumor-cytotoxicity without harming the normal tissue (16, 17). The main promise of immunotherapy is not only to combat tumor growth by eliminating cancer cells, but to keep an army of memory cells to avoid tumor recurrence, a facet of treatment that will be crucial for GBM (18). The discovery of specific tumor associated peptides presented by major histocompatibility complexes (MHC) (19, 20); and inhibition of immune checkpoint molecules (cytotoxic T lymphocyte antigen 4 (CTLA4) and programmed cell death 1 (PD1) that regulate T cell activation; opened new doors for the treatment of cancer, by augmenting the natural functions of the immune system (17, 21–23). Biological options of immune-based therapies that have been developed include checkpoint inhibitors, cellular therapies, vaccines, engineered T cells, small peptide inhibitors of specific pathways, monoclonal antibodies, and cytokine therapy (24–26).

Currently there are no Food and Drug Administration (FDA) approved immunotherapy regimens for the treatment of GBM (16). Although there are several immune-based therapies currently being tested for GBM, the majority evaluate mainly tolerance and toxicity (16). Even while some immunotherapies have shown promising clinical results when evaluated as a monotherapy, their true impact when combined with, or given alongside of, standard of care is unknown (27). Moreover, only a few of these modalities have progressed to the phase II or III clinical trial setting to systematically test their impact on overall survival, progression of the disease, and severe adverse events when administered in combination with current standard of care (28). Because of this, little is known about the true clinical benefits and toxicity profile of immunotherapy given in combination with chemo-radiotherapy. Ongoing (unpublished) phase II or III clinical trials of immune checkpoint inhibitors used in combination with standard of care for newly diagnosed GBM failed to meet survival expectancy and PFS in MGMT methylated (CheckMate 548, NCT02667587) (29, 30) or un-methylated (CheckMate 498, NCT02617589) (31, 32) GBM patients. The randomized trial phase II/III NRG-BN007 evaluating ipilimumab and nivolumab versus temozolomide to radiotherapy in un-methylated GBM patients is also ongoing, and the results are awaited with high expectations (33). Unfortunately, most of the results with different immunotherapies in GBM have disappointed the medical community in regards of improving survival for these patients. However, these studies are essential to understand the benefits and the associated risks of immunotherapies in gliomas.

This gap in the knowledge could be masking the full potential of immunotherapy when used as an adjuvant treatment in GBM and can be limiting our ability to make fully informed decisions in regard to adding immunotherapy to the current standard of care for GBM. To address the knowledge gap in this area and to provide a scientific rationale about the possible synergistic effect that chemo-radiation and immunotherapy may have when used together, we performed a systematic review and meta-analysis. We analyzed and compared 1-year overall survival (OS), 1-year progression free survival (PFS) and grade 3 to 5 adverse events, in the immunotherapy plus chemo-radiotherapy regimen (defined as immunotherapy and standard of care or combined group), *versus* chemo-radiotherapy alone regimen (defined as standard of care or control group), in patients with newly diagnosed GBM.

## Materials and Methods

### Study Selection Criteria

A search strategy was developed using the Population, Intervention, Comparison, Outcome, Study type (PICOS) question format: In newly-diagnosed glioblastoma patients (Population) that receive standard of care with or without immunotherapy (Intervention and Comparison), what are the overall survival, progression free survival and severe adverse events grades 3 to 5 (Study Type) based on results from phase II and III clinical trials (Study type) (**Supplementary Table 1**).

**Figure 1:**
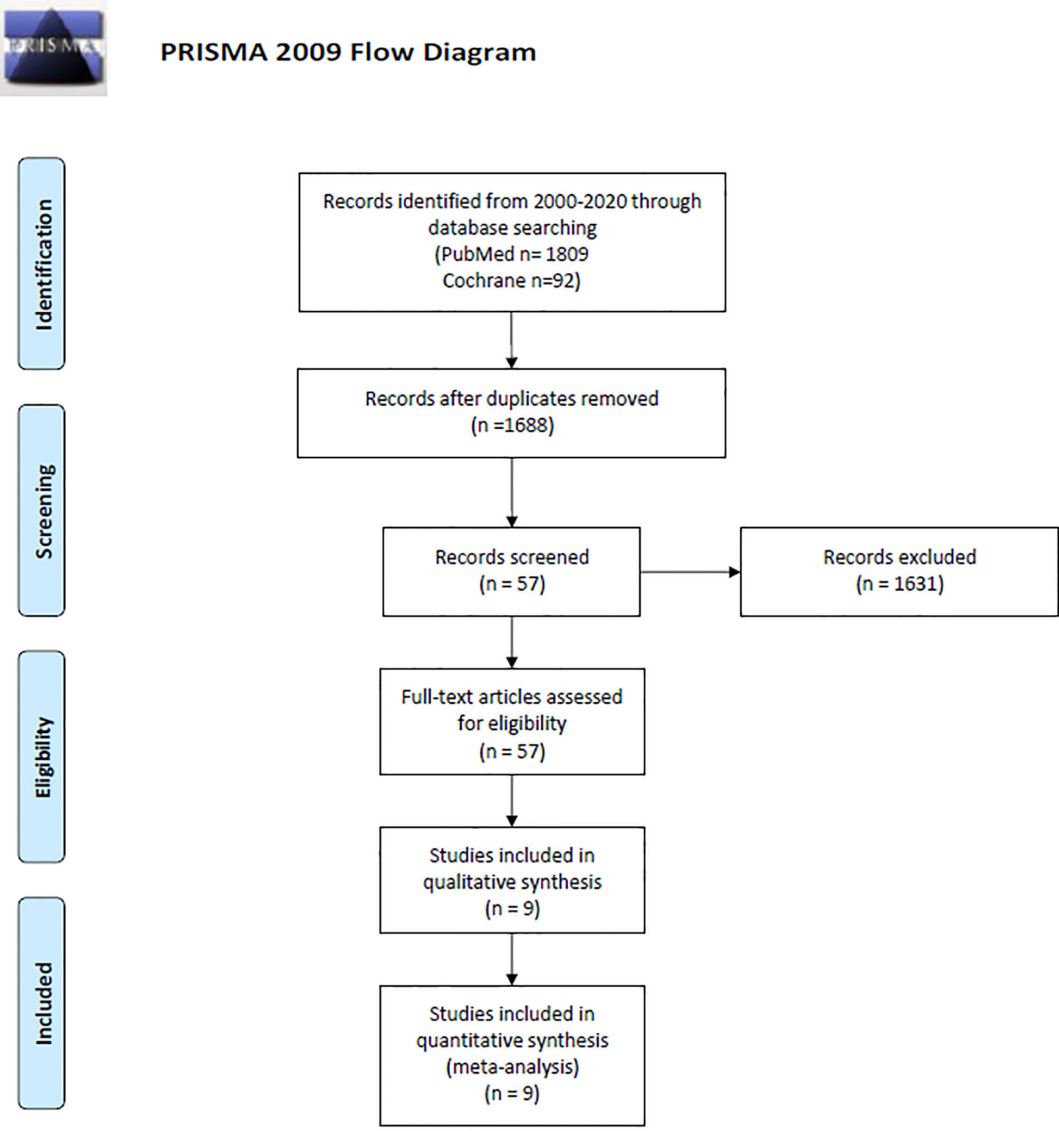
PRISMA diagram describing study design and selection (34).

A literature search in PUBMED and Cochrane was performed by three independent reviewers according to the Preferred Reporting Items for Systematic Reviews and Meta-Analyses (PRISMA) (**Figure 1** and **Supplementary Table 2**) **(** 34) and Meta-Analyses and Systematic Reviews of Observational Studies (MOOSE) (35) **(** **Supplementary Table 3** **)** guidelines. Our search included phase II and/or III clinical trials, published from January 1st 2000 to October 1st 2020. The terms used for literature search included “glioblastoma,” OR “newly diagnosed glioblastoma,” OR “malignant glioma,” AND “radiation” AND “chemotherapy” OR “radio-chemotherapy,” AND “immunotherapy”. These terms were specifically used as follows in batch searches across both databases: glioblastoma and radio chemotherapy and immunotherapy, newly diagnosed glioblastoma and radio chemotherapy and immunotherapy, newly diagnosed glioblastoma and radio chemotherapy, newly diagnosed glioblastoma and immunotherapy, glioblastoma and immunotherapy, newly diagnosed glioblastoma and radiotherapy, newly diagnosed glioblastoma and chemotherapy and malignant glioma and radio chemotherapy and immunotherapy. Any disagreement was discussed and resolved by the reviewers and senior author. All the articles resulting from our initial search were analyzed for our inclusion and exclusion criteria (**Table 1**) and only articles that satisfied all our inclusion and exclusion criteria were selected for the final analysis.

**Table 1:** Inclusion and exclusion criteria.

Inclusion	Exclusion
(1) patients with newly diagnosed GBM confirmed by pathology	(1) patients with recurrent GBM
(2) with no other neurological diseases	(2) with other neurological diseases
(3) study specifically described as clinical phase II or III	(3) study not specified as clinical phase II or III
(4) with two arm groups: control and combined	(4) with one arm group only
(5) published only in English	(5) published in other language different than English
(6) limited to human subjects	(6) not limited to human subjects

### Data Extraction

All data were extracted from the main manuscript and supplementary text, tables, and figures of the included articles. Our primary outcome of interest was clinical efficacy of combined therapy compared with control therapy in patients with newly diagnosed GBM; (as reflected by median and 1-year survival and PFS). Estimation of survival and PFS at 1 year was done by using the software Plot Digitizer^®^ v.2.6.8 in those articles that did not report these parameters as such. OS and PFS were calculated from the data based on date of surgery until event (death or recurrence). The secondary outcome was toxicity, measure as the number and proportion of incidental grades 3 to 5 adverse events by the end of the study in the standard of care and immunotherapy group as compared with the standard of care group alone. Two independent investigators extracted the data (M.L.V and K.M.B), and confirmation of extracted data was done by two other independent reviewers (H.R.G and J.M.S).

### Statistical Analysis

R Studio (36), Version 1.1.383 (Boston, MA) was used to perform the statistical analyses. Generation of random-effects meta-analyses, assessments of heterogeneity and publication bias, and generation of forest plots were conducted as previously described by Lehrer et al. (37) Study arms were compared using the Wald-type test, where the null-hypothesis was rejected for p<0.05.

### Quality Evaluation of Clinical Trials

Publications accepted in the study were methodologically evaluated according to the revised Cochrane risk-of-bias tool for randomized trials (38). This system categorizes the studies in low, unclear or high risk of bias, according to the following parameters: random sequence generation, allocation concealment, blinding of participants and personnel, blinding of outcome assessment (self-reported outcomes and objective measures), incomplete outcome data, selective reporting and other bias. Buchroithner, Cho, Kong, Ursu, and Wakabayashi trials had unclear to high risk of bias in at least three different parameters evaluated, Wheeler and Sampson had high risk of bias in five parameters. Weller and Wen trials had unclear risk of bias in only one parameter, making them the trials with lower risk of bias in the entire study **(** **Supplementary Figure 1** **).**

We also evaluated the quality of the included trials with the Grading of Recommendations, Assessment, Development, and Evaluation (GRADE) system (GRADEpro). For the evaluated outcomes, 1-year OS and PFS were rated with moderate quality, whereas SAEs grades 3 to 5 were rated as low quality **(** **Supplementary Tables 4** and **5** **).**

## Results

### Study Characteristics

Our initial search resulted in 1901 publications (1809 from PUBMED and 92 from Cochrane). Due to duplicity 213 articles were eliminated, while 1631 were eliminated after the title/abstract was screened based on our inclusion and exclusion criteria. A total of 57 papers met our initial screening of the eligibility criteria. The full text of those articles was analyzed and a total of nine articles met all our inclusion and exclusion criteria and were included for final analysis. Of the nine studies included, seven were phase II clinical trials (77.7%) and two were phase III (22.2%) (**Figure 1** and **Table 2**). Three studies were conducted in North America (39–41), two in Europe (42–44), three in Asia (45–47), and one in Europe and North America (48). Combining all the studies, a total of 1239 patients were included. There were 583 (47%) patients in the immunotherapy and standard of care group, and 656 (52.9%) in the standard of care group. Of the seven articles that described gender information of the included patients (40, 42–50), 619 (60.5%) were male and 403 (39.4%) were female; two articles did not include this information (39, 41). Average age of the patients ranged from 27 years to 72.2 years. Regarding the immunotherapy approach, four studies used cellular vaccines, two used peptide vaccines, one used immunostimulating oligodeoxynucleotides, one interferon β, and one gene-mediated cytotoxic immunotherapy. All trials combined one of these regimens in combination with chemoradiotherapy, while a chemoradiotherapy regimen alone group was used as control in all of them. Four immune based approaches were administered intravenously (44.4%), one cranially (11.1%), three intradermally (33.3%), and one intracranially, intravenously and/or orally (11.1%) (**Supplementary Table 6**).

**Table 2:** Overall characteristics of the studies.

First Author	Buchroithner	Cho	Kong	Sampson	Ursu	Wakabayashi	Weller	Wen	Wheeler	Total	Median
**Year**	2018	2011	2017	2010	2017	2018	2017	2019	2016		
**Trial Phase**	II	II	III	II	II	II	III	II	II		
**Country of Publication**	Austria	China	Korea	USA	France	Japan	Switzerland/USA	USA	USA		
**Age (low limit)**	19	14	19	29	42	22	51	22	32		**27**
**Age (high limit)**	70	70	69	71	78	75	64	81	72		**72.2**
**Patients Included (n)**	76	34	180	35	81	122	405	124	182	**1239**	
**Immunoteraphy group (n)**	34	18	91	18	39	59	195	81	48	**583**	
**Immunotherapy group (%)**	44.73684211	52.94117647	50.55555556	51.42857143	48.14814815	48.36065574	48.14814815	65.32258065	26.37362637		**47**
**Control group (n)**	42	16	89	17	42	63	210	43	134	**656**	
**Control group (%)**	55.26315789	47.05882353	49.44444444	48.57142857	51.85185185	51.63934426	51.85185185	34.67741935	73.62637363		**52.9**
**Immunotherapy type used**	Tumor lysate-charfed autologous dendritic cells (Audencel)	Whole-cell lysate dendritic cell vaccine	Autologous cytokine- induced killer cells	PEPvIII vaccine (13-amino acid peptide w/ an additional terminal cystine that spans the EGFRvIII mutation)	Immunostimulating oligodeoxynucleotides containing unmethylated cytoside-guanosine motifs (CpG-ODN)	Interferon β	Rindopepimut	ICT-107 (autologous dendritic cells)	Aglatimagene besadenovec (AdV- tk) plus valcyclovir (gene-mediated cyottoxic immunotherapy)		
**Male (n)**	51	16	102	NA	48	73	254	75	NA	**619**	
**Male %**	67.10526	47.05882	56.66667	NA	59.25926	59.83607	62.71605	60.48387	NA		**60.5**
**Female (n)**	25	18	78	NA	33	49	151	49	NA	**403**	
**Female %**	32.89474	52.94118	43.33333	NA	40.74074	40.16393	37.28395	39.51613	NA		**39.4**

Finally, we analyzed the clinical trials that used the same immunotherapy strategy. The cellular vaccines subgroup was the only category with enough studies to perform a statistical analysis (four studies total: Buchroithner, Wen, Cho, and Kong) (40, 42, 45, 46).

### Clinical Outcomes

#### Combined Therapy Was Not Associated With Significant Improvement in Overall Survival

**Figure 2:**
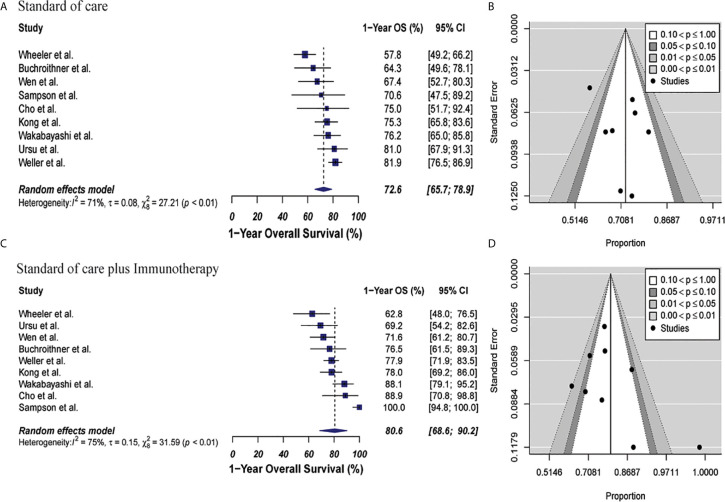
Meta-analysis of 1-year OS in patients from all trials receiving standard of care or immunotherapy plus standard of care therapy. **(A, B)** 1-year OS forest and funnel plots of patients who received standard of care treatment [72.6% (95% CI: 65.7%–78.9%, I2: 71%)]. **(C, D)** 1-year OS forest and funnel plots of patients who received standard of care and immunotherapy treatment [80.6% (95% CI: 68.6%–90.2%, I2: 75%)] (p = 0.15). Funnel plots showing no significant publication bias found in the present meta-analysis in both groups with p-values of 0.89 and 0.72, in standard of care and immunotherapy treatment group respectively. In forest plots size of each square is proportional to its corresponding study’s total sample size. The ends of the horizontal bars denote a 95% CI. The diamond gives the overall odds ratio for the combined results of all trials. The center denotes the odds ratio, and the extremities denote the 95% CI.

The 1-year OS was 72.6% (95% CI: 65.7%–78.9%, I2: 71%) versus 80.6% (95% CI: 68.6%–90.2%, I2: 75%) for the control and combined therapy groups, respectively (p = 0.15). Publication bias was absent with p-values of 0.89 and 0.72, respectively. These results are shown in **Figure 2**. Median OS was 16.9 months in control group vs. 20.1 months in the combined therapy group (**Supplementary Table 7**).

**Figure 3:**
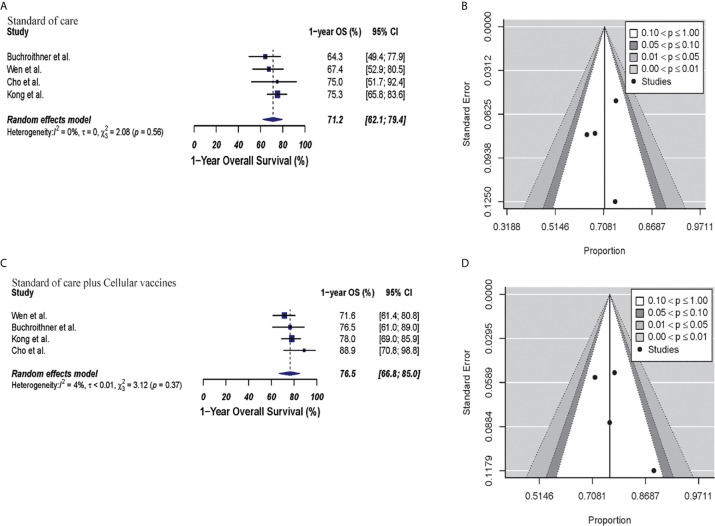
Meta-analysis of 1-year OS in patients from trials receiving standard of care or cellular vaccine therapy plus standard of care therapy. **(A, B)** 1-year OS forest and funnel plots of patients who received standard of care treatment [71.2% (95% CI: 62.1–79.4, I^2^ = 0%)]. **(C, D)** 1-year OS forest and funnel plots of patients who received standard of care and cellular vaccine treatment [76.5% (95% CI: 66.8%–85%, I^2^ = 4%) (p = 0.21)]. Funnel plots showing no significant publication bias found in the present meta-analysis in both groups with p-values of 0.82 and 0.36, in standard of care and immunotherapy treatment group respectively. In forest plots size of each square is proportional to its corresponding study’s total sample size. The ends of the horizontal bars denote a 95% CI. The diamond gives the overall odds ratio for the combined results of all trials. The center denotes the odds ratio, and the extremities denote the 95% CI.

In the cellular vaccine subgroup analysis, 1-year OS was 71.2% (95% CI: 62%.1–79.4%, I^2^ = 0%) *versus* 76.5% (95% CI: 66.8%–85%, I^2^ = 4%) for the standard of care and immunotherapy groups, respectively (p = 0.21). There was no publication bias with p-values of 0.82 and 0.36, respectively. These results are shown in **Figure 3**.

#### Combined Therapy Was Not Associated With Significant Improvement in Progression Free Survival

**Figure 4:**
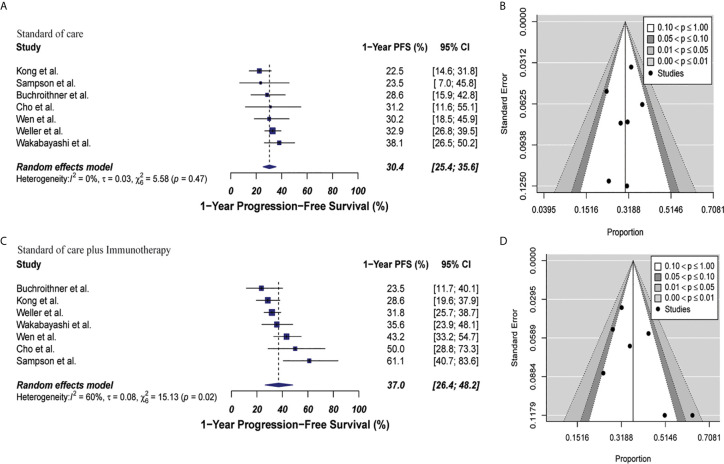
Meta-analysis of 1-year PFS in patients from all trials receiving standard of care or immunotherapy plus standard of care therapy. **(A, B)** 1-year PFS forest and funnel plots of patients who received standard of care treatment [30.4% (95% CI: 25.4–35.6; I^2^ = 0%)]. **(C, D)** 1-year PFS forest and funnel plots of patients who received standard of care and immunotherapy treatment [37% (95% CI: 26.4–48.2, I^2^ = 60%) (p = 0.17)]. Funnel plots showing no significant publication bias found in the present meta-analysis in both groups with p-values of 0.63 and 0.13 in standard of care and immunotherapy treatment group respectively. In forest plots size of each square is proportional to its corresponding study’s total sample size. The ends of the horizontal bars denote a 95% CI. The diamond gives the overall odds ratio for the combined results of all trials. The center denotes the odds ratio, and the extremities denote the 95% CI.

The estimated 1-year PFS was 37% (95% CI: 26.4–48.2, I^2^ = 60%) versus 30.4% (95% CI: 25.4–35.6; I^2^ = 0%) for the combined therapy and control groups, respectively (p = 0.17). Publication bias was absent with p-values of 0.13 and 0.63, respectively **(** **Figure 4** **)**. Two clinical trials were not included in this analysis since they did not describe PFS information properly for GBM patients (35, 44). Median PFS were 8.5 months and 7.7 months in the combined therapy group and control group, respectively (**Supplementary Table 7**).

**Figure 5:**
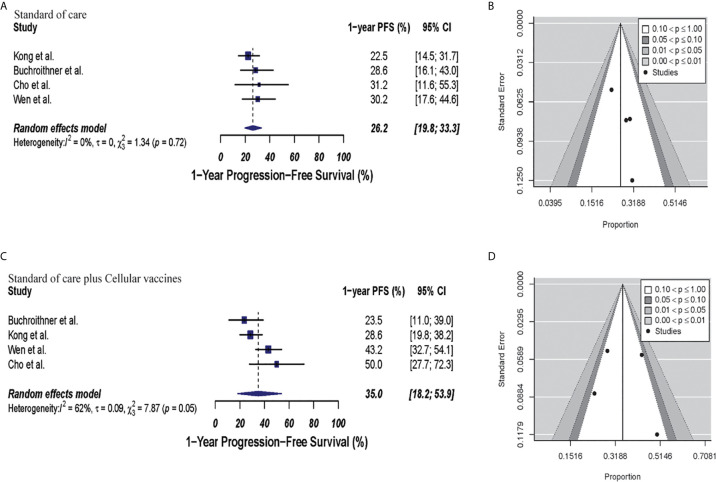
Meta-analysis of 1-year PFS in patients from trials receiving standard of care or cellular vaccine therapy plus standard of care. **(A, B)** 1-year PFS forest and funnel plots of patients who received standard of care treatment [26.2% (95% CI: 19.8–33.3, I^2^ = 0%)]. **(C, D)** 1-year PFS forest and funnel plots of patients who received standard of care and cellular vaccine treatment [35% (95% CI: 18.2–53.9, I^2^ = 62%) (p = 0.19)]. Funnel plots showing no significant publication bias found in the present meta-analysis in both groups p = 0.27 and p = 0.73, in standard of care and immunotherapy treatment group respectively. In forest plots size of each square is proportional to its corresponding study’s total sample size. The ends of the horizontal bars denote a 95% CI. The diamond gives the overall odds ratio for the combined results of all trials. The center denotes the odds ratio, and the extremities denote the 95% CI.

In the cellular vaccine subgroup analysis, 1-year PFS was 35% (95% CI: 18.2–53.9, I^2^ = 62%) versus 26.2% (95% CI: 19.8–33.3, I^2^ = 0%) in the combine therapy group and control group, respectively (p = 0.19). No publication bias was present in neither of both groups (p = 0.73 and p = 0.27, respectively) as described in **Figure 5**.

#### Combined Therapy Was Not Associated With Significant Increase in Incidence of Grade 3-5 Severe Adverse Events

**Figure 6:**
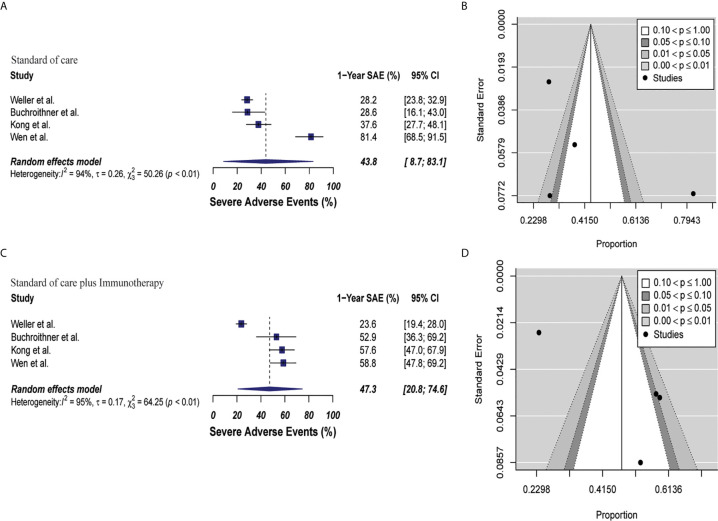
Meta-analysis of SAEs grade 3 to 5 in patients from trials receiving standard of care or immunotherapy plus standard of care therapy. **(A, B)** SAEs forest and funnel plots of patients who received standard of care treatment [43.8% (95% CI: 8.7–83.1, I^2^ = 94%)]. **(C, D)** SAEs forest and funnel plots of patients who received standard of care and immunotherapy treatment [47.3% (95% CI: 20.8–74.6, I^2^ = 95%) (p = 0.81)]. Funnel plots showing no significant publication bias found in the present meta-analysis in both groups p = 0.37 and p = 0.22, in standard of care and immunotherapy treatment group respectively. In forest plots size of each square is proportional to its corresponding study’s total sample size. The ends of the horizontal bars denote a 95% CI. The diamond gives the overall odds ratio for the combined results of all trials. The center denotes the odds ratio, and the extremities denote the 95% CI.

Toxicity analysis was performed with data from four clinical trials that described SAE grades 3 to 5 appropriately (40, 42, 46, 48). SAE was 47.3% (95% CI: 20.8–74.6, I^2^ = 95%) *versus* 43.8% (95% CI: 8.7–83.1, I^2^ = 94%) in the combined therapy group and control group, respectively (p = 0.81). There were not bias associated with publication in these studies (p = 0.22 and p = 0.37, respectively). These results are depicted in **Figure 6**. Of the 1110 pooled patients included in this analysis, 184 (16.5%) in the control group versus 201 (18.1%) patients in combined therapy group had a SAE (**Supplementary Table 8**).

**Figure 7:**
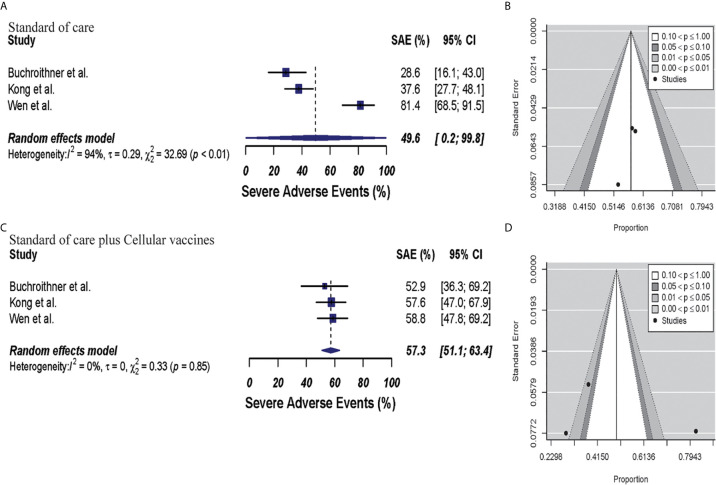
Meta-analysis of SAEs grade 3 to 5 in patients from trials receiving standard of care or cellular vaccine therapy plus standard of care. **(A, B)** SAEs forest and funnel plots of patients who received standard of care treatment [49.6% (95% CI: 0.2–99.8, I^2^ = 94%)]. **(C, D)** SAEs forest and funnel plots of patients who received standard of care plus cellular vaccine treatment [57.3% (95% CI: 51.1–63.4, I^2^ = 0%) (p = 0.68)]. Funnel plots showing no significant publication bias in both groups with p-values of 0.78 and 0.19 in standard of care and immunotherapy treatment group respectively. In forest plots size of each square is proportional to its corresponding study’s total sample size. The ends of the horizontal bars denote a 95% CI. The diamond gives the overall odds ratio for the combined results of all trials. The center denotes the odds ratio, and the extremities denote the 95% CI.

In the cellular vaccine subgroup analysis, one trial did not include a full description of SAE (45); thus, subgroup analysis was performed with the other three studies (40, 42, 46). SAEs analysis revealed higher occurrence in the vaccine and standard of care group (57.3%, 95% CI: 51.1–63.4, I^2^ = 0%) when compared to standard of care group (49.6%, 95% CI: 0.2–99.8, I^2^ = 94%), p = 0.68. Publication bias was absent with p-values of 0.19 and 0.78, respectively. These results are shown in **Figure 7**.

## Discussion

In this systematic review and meta-analysis, we objectively analyzed the survival, progression free survival, and toxicity profile of immunotherapy in combination with chemo-radiation versus chemo-radiotherapy alone in newly diagnosed GBM. In regard to overall survival and progression free survival we found that immunotherapy marginally prolonged both; however, this effect was not statistically significant. We also found that immunotherapy with standard of care does not increase grade 3 to 5 SAE in a statistically significant manner when compared to standard of care alone **(** **Table 3** **)**.

**Table 3:** Summarized results for 1-year OS and PFS, and SAEs in standard of care and standard of care and immunotherapy groups, and for trials that used cellular vaccines only.

Group	Outcome	Wald Test	Peter’s Test
Control	1y OS	Ref	0.89
Combined	1y OS	0.15	0.72
Control (Vaccine Only)	1y OS	Ref	0.82
Combined (Vaccine Only)	1y OS	0.21	0.36
Control	1y PFS	Ref	0.63
Combined	1y PFS	0.17	0.13
Control (Vaccine Only)	1y PFS	Ref	0.27
Combined (Vaccine Only)	1y PFS	0.19	0.73
Control	SAE	Ref	0.37
Combined	SAE	0.81	0.22
Control (Vaccine Only)	SAE	Ref	0.78
Combined (Vaccine Only)	SAE	0.68	0.19

As inherent in the nature of a meta-analysis, our overall analysis is limited by the individual limitations of each of the studies included in the analysis. The number of patients treated in each trial varied widely, from 34 patients (45) to 405 patients (48), between trials thus weighing differently on the overall analysis.

The immunotherapy was administered in different ways [intravenous, intradermal, oral (39, 40, 42, 45–48) and intracerebral (41, 44)] possibly limiting therapeutic distribution as well as immune cell recruitment and diminished effector function. Furthermore, the targeted dose, as well as the number of doses administered, varied among trials. Although most of the studies administered the immunotherapy agent at minimum two to five times, the total number of doses was patient specific and was dependent on clinical progression or death. There was also a variability in the follow-up period between trials, making the comparison between trials heterogeneous. The timing of when the immunotherapy was initiated varied between trials which could influence the overall toxicity profile of the combined strategy and finally determined the reported SAEs in the trials. Most of the trials used an early start of immunotherapy including: 1 to 2 weeks after chemoradiation (42, 48), at the same time as chemoradiation (46), preoperatively (41, 44), on alternate days during radiation (47), and between radiotherapy and at the beginning of temozolomide (40). While two studies showed a mid-late start of immunotherapy: within 6 weeks of completing radiation (39), and 1 to 2 months postoperatively (45).

There was significant variation among concomitant and maintenance therapy regiments that can directly influence OS and PFS of the individual studies. While most of the studies (6/10) used the standardized dose of TMZ and radiation during concomitant treatment (radiation at a dose of 60 Gy and TMZ at a dose of 75 mg/m^2^), two studies did not report doses (41, 44), and one study (45) used a higher TMZ dose (100 mg/m^2^). During the maintenance phase, TMZ was the most used drug, with some variabilities in dosage (100 mg/m^2^ to 200 mg/m^2^) and treatment length. Other agents used as maintenance treatment were: bevacizumab, nitrosoureas, irinotecan, tumor-treating fields, other chemotherapy (not defined), investigational drugs, tyrosine kinase inhibitors and check-point inhibitors (42, 44). In addition, there is significant variation in the radiation therapy practices for GBM around the word in terms of radiation dose and treatment field (51–53). Since the clinical trials included in our analysis originated in several different countries this variability is built into our model and is likely playing a critical role in the overall heterogeneity of the studies and overall outcome parameters being assessed.

Another known factor that significantly impacts patient’s overall survival and PFS is the extent of resection (54, 55), which varied widely amongst the studies included in this meta-analysis. The variability in the total volume of tumor resected between studies is a limiting factor in evaluating the true response rate of immunotherapy. Patients were categorized under gross, subtotal, partial, and large resection, and in residual or non-residual disease. To make our analysis as consistent as possible, our calculations in the studies that considered those variables, were based on OS and PFS of total number of patients included without making distinctions in the surgical outcome. However, this almost certainly introduced some variability and noise into our data that could have been accounted for with a more consistently designed set of trials. Also, PFS assessment variability between studies has to be considered, since pseudo-progression is still a controversial topic (56) that need to be addressed to ensure clear accountability of this parameter in response to immunotherapy. Although the Macdonald criteria for tumor response assessment was used by the majority of the studies, this classification has multiple limitations such as the presence of necrosis or residual changes secondary to tumor resection. As a result, a more comprehensive imaging criteria was defined for assessing response/progression by the Response Assessment in Neuro-Oncology (RANO) (57) and the immunotherapy response assessment for Neuro-oncology (iRANO). The RANO criteria is widely used in clinical trials in oncology for an accurate assessment of pseudo progression in response to temozolomide and radiotherapy in malignant gliomas during concomitant or maintenance regimens (58). RANO criteria does not suit properly the needs for response evaluation in patients treated with immune based therapies. Thus, the iRANO criteria was defined to assess clinical outcomes and tumor regression despite progression of the disease in the context of immunotherapy (59). It accounts for the differential mechanistic and imaging findings elicited by immunotherapy to the ones by chemoradiation; such as enhancing lesions outside the main radiation field and delayed therapeutic efficacy, that with other criteria would be classified as disease progression (60). iRANO defines disease progression when tumor persistence is registered in a specific period of time, after an initial radiographical evidence of tumor progression in response to immunotherapy (60).

Finally, although for the purposes of this analysis, we combined the included studies into the broad umbrella of “immunotherapy” it is useful to examine the specific therapies in more detail to clearly understand the clinical immunotherapy landscape.

### Cellular Vaccines

Our analysis included three phase II clinical trials (Buchroithner et al. with 76 patients, Cho et al. with 34 patients and Wen et al. with 124 patients), that used dendritic cells, and one phase III trial (Kong et al. with 180 patients) that used cytokine-induced killer (CIK) cells with standard of care. Of these, the trial by Cho et al. showed the highest improvement in median and 1-year survival expectancy (median OS:15 *vs* 31.9 months, and 1-year OS: 75% *vs* 88.9%, in control *versus* combined therapy, respectively). This can be attributed primarily to two distinct features of the trial: 1) the strategy of using personalized DCs vaccines, where a diverse group of individualized highly immunogenic peptides educating the DCs, could potentially elicit a better tumor clearance by the immune system and 2) the adjuvant treatment strategy used upon tumor recurrence, defined by tumor size increase >20%, which included repeated surgical-intervention, chemotherapy or boost gamma knife radiosurgery. In addition, a reinforced dose of vaccination (made from recurrent tumor tissue) was given to patients that underwent a second surgery (6 of 18 patients).

Of the four trials in this category the trial by Kong et al. was a phase III trial that showed very modest effect with an extension of 5.6 months in median OS and 3% improvement at 1-year survival. The limited response in survival in this study could be mainly due to biodistribution of the cytokine induced lymphocytes and their ability to selectively home into the tumor microenvironment. Additionally, there was some variability in the timing of the administration of the cells which most likely influenced the overall outcome of the trial.

### Peptide Vaccines

EGFR (epidermal growth factor receptor) overexpression is one of the most prevalent mutations in GBM (60%) (61). 20% to 30% of these tumors, present a deletion of exons 2 to 7 in the EGFRvIII receptor (type III EGFR) (62). Sampson et al, phase II trial included 35 patients using EGFRvIII-targeted peptide vaccine and chemo-radiotherapy, showed an extension of approximately 9 months in median OS and PFS, extension of almost 30% at 1-year survival and 40.1% at 1-year PFS. However, in the phase III clinical trial that included 405 patients using this strategy, Weller et al, found no benefits in overall survival or PFS. (Median OS of ˜ 20 months in both groups, 1-year survival decreased by 4% with immunotherapy, improvement of 0.6 months in median PFS and 1% decrease at 1-year PFS, with combined treatment). Benefits in the trial by Sampson et al. could be due to an enhanced humoral and cellular immune response rate produced by the vaccine elicited with dose intensified TMZ regiment (100 mg/m^2^ for first 21 days of 28 days cycle) in comparison with standard dosing (200 mg/m^2^ for first 5 days of 28 days cycle), that was significant despite chemotherapy-induced lymphopenia. In comparison the phase III trial by Weller et al. used only the standard TMZ protocol and did not have the dose intensified arm. It is well described that the patient selection criteria and overall health status of the patients enrolled heavily weighs on the overall outcome of the patients in clinical trial (63). The majority of the patients included in the Sampson et al. trial had KPS score of 100 whereas majority of the patients in the Weller et al. trial had RPA class IV or higher, which most likely influenced the overall outcome. Most importantly the trial by Sampson et al. used historical control group which might not truly capture the complexity of the trial group and hence does not represent a true control arm.

### Other Immune-Based Therapies

Other forms of immunotherapy clinical trials included: a) intracerebral administration of CpG ODN (44), b) intravenous IFNβ (47) and c) gene-mediated cytotoxic immunotherapy (aglatimagene besadenovec (AdV-tk), an adenoviral vector containing the herpes simplex virus thymidine kinase gene, followed by an antiherpetic prodrug such as valacyclovir) (41).

The phase II trial by Ursu et al. included 81 patients, showed no improvement in median and 1-year survival or median PFS with intracerebral administration of CpG ODN and chemo-radiation. (median OS ˜ 18 months and median PFS ˜ 9 months in both groups, and 1-year survival decreased by 10%). The results of this trial again highlight the difficulties surrounding the issue of drug delivery and penetration in the context of GBM. In this trial CpG ODN was injected by needles in the resection cavity which restricted the amount of drug that penetrated beyond a small perimeter. Most local drug delivery studies report reliable penetration only few millimeters from the site of injection (50), whereas studies with convention enhanced delivery have reported a diffusion in centimeters (64, 65), still any tumor cells beyond that margin will not be affected by this treatment strategy.

The phase II clinical trial by Wakabayashi et al. included 122 patients and evaluated IFNβ in combination with standard of care for newly diagnosed GBMs and found an extended median OS of almost 4 months (1-year survival rate improved by 11%) with combination therapy but a decrease in PFS by 1.6 months (1-year PFS decreased by 2.5%). IFNβ was used as a chemosensitizer that enhances the toxicity of chemotherapeutic agents such as TMZ. Thus, it is not surprising that the combination therapy arm had significantly higher rate of hematological as well as non-hematological toxicities. Higher toxicity also negatively impacted treatment compliance to the point that a high number of patients terminated the treatment protocol prematurely, which most likely played a role in the poor overall survival.

The Wheeler et al. phase II trial included 182 patients total and tested gene-mediated cytotoxic (GMC) immunotherapy in combination with standard of care. This trial did not yield survival benefits either, GBM data sub analysis showed 3-month prolonged median OS and 1-year survival rate improved by 5% that were not statistically significant. Efficacy and biodistribution of the virus delivery by local injection in the resection cavity is limited by the previously outlined constrains of drug or biological agents penetration issues in the CNS microenvironment. In addition, virus particles induce prompt and effective anti-virus immune response which accelerates the clearance of virus further limiting its penetration in the tumor microenvironment (66). If the issue of immune mediated virus clearance is not weighted in properly into the timing of administration of the activating pro-drug there will be no effective immune response. Extent of resection is also important when assessing the likelihood of overall success of immunotherapy since immunosuppressive features such as expression of PD-L1, presence of regulatory cells etc. in the residual tumors decreases the overall efficacy of the immunotherapy (67).

In summary, upon closer evaluation of the individual immunotherapies across the trials analyzed in this study, it becomes clear that there is a large amount of variability in patient response within the trials and across the trials. This highlights the often-seen tail phenomenon of GBM patients in immunotherapy trials where there are small groups of patients who do respond and do while even if the majority of patients in cohort may not benefit resulting in an overall negative trial (16). This data points toward the need for better design of immunotherapy clinical trials that include potential responders using synergistic combination therapy that boosts the overall function of the immune system in addition to tumor specific immune response.

### Severe Adverse Events in Immunotherapy

SAEs in clinical trials are considered as complications/toxicity, morbidity or mortality as a result of a tested treatment (68). They can be symptomatic (reported by the patient) or asymptomatic (detected during a physical examination, laboratory results or imaging reports) (69). Grade 1 and 2 events are mild or moderate, or even asymptomatic symptoms that can be managed with outpatient medication. Grade 3 events are severe non-immediately-life threatening symptoms that can be controlled usually during inpatient treatment or prolonged hospitalization (parental administration of drugs or surgical intervention). Grade 4 events put the life of the person at risk, and can result in disabilities and organ dysfunctions, whereas grade 5 events are deadly (69, 70).

There was significant heterogeneity in the SAE reporting criteria used by each of the clinical trials included in this meta-analysis. Criteria used for the grading and defining grade 3 to 5 SAEs in the studies included in this analysis were the National Cancer Institute (NCI) Common Terminology Criteria for Adverse Events (CTCAE) version 3.0 by Wheeler et al, Wakabayashi et al, and by Kong et al, and version 4.0 by Weller et al. The precursor of this classification, the NCI Common Toxicity Criteria (CTC) version 2.0 was used by Sampson et al, version 3.0 by Ursu, version 4.0 by Buchroithner, and 4.03 by Wen. Cho’s trial did not specify the classification used. Analysis of SAEs grade 3 to 5 in Cho, Sampson, Ursu, Wakabayashi and Wheeler’s trials were not possible due to the presentation of the data. (ie, grades 2 and 3 events reported combined, events non-separated by grade, and events reported separately during concomitant and/or maintenance treatment).

We only used four out of the nine studies for the grade 3 to 5 SAE analysis (40, 42, 46, 48). Our analysis showed an increased occurrence of grade 3 to 5 SAEs associated with immunotherapy in combination with chemo-radiation compared to chemo and radiation but the effect failed to reach statistical significance. Although some of these events may be expected due to the immune nature of the therapies and the known effects of chemoradiation, the majority of these were severe non-immediately-life threatening SAEs controlled with inpatient medication or hospitalization. The only two deadly events found with our analysis was with the immunotherapy CpG in the study by Ursu et al. (secondary to reactivation of hepatitis B infection) and the peptide vaccine in Sampson’s trial (due to pulmonary embolism). Among immunotherapy approaches, the most common SAEs were: headache, nausea, vomiting, seizures, constipation, diarrhea, weakness, anorexia, pyrexia, increase transaminases, increase lipases, increase intracranial pressure, and rash/allergic reactions. Hematological toxicities frequently seen after immunotherapy and standard of care were: lymphopenia, thrombocytopenia and neutropenia (For grading criteria of the most common grade 3 to 5 SAEs found among trials go to **Supplementary Table 9**).

Pneumonia and acute-renal failure were specifically described in one trial using cellular vaccines (46). Peptide vaccines were specifically linked with brain edema, and one deadly event due to pulmonary thromboembolism (48). CpG ODN and chemo-radiation was related with post-surgical hematoma, seizures (probably secondary to the intracerebral administration of the agent), and with one death related with reactivation of hepatitis B infection (although a full analysis of SAEs was not possible due to the format used for data presentation). Gene-mediated cytotoxicity therapy was related with hemiparesis (motor neuropathy), speech impairment, insomnia, and wound complication (specific analysis of SAEs 3 to 5 was not possible due to how the data was presented).

Taken together, this evidence demonstrates that although adverse events grade 3 to 5 are more frequent in the immunotherapy and chemo-radiation treatment when compared with chemo-radiation only, they are mostly non-deadly toxicities that can be managed during an inpatient encounter or hospitalization. Importantly, our findings are in accordance with the data described in the retrospective study of 22 trials done by Magee and colleagues in 2020, where the risk of an adverse event grade 3 or higher was increased in the immunotherapy arm compared with the chemotherapy arm alone for solid tumors (71). Nonetheless, our data show that this trend in increased toxicity in immunotherapy was not statistically significant.

## Strengths and Limitations

The strength of our study lies in the strict inclusion and exclusion criteria used for article selection. We used a clearly defined list formed by several characteristics allowing us to compare control versus combined therapy in newly diagnosed GBM patients. This eliminated the potential of patients having received prior treatment. Additionally, we included phase II and III clinical trials that evaluated survival benefits (OS and PFS), as well as toxicity (SAEs) secondary to treatment. By doing so, we were able to perform a more reliable comparison of the results in the control and combined groups for each study. Moreover, we attempted to reduce heterogeneity by excluding trials that were not specifically described as phase II or III, even if they described OS, PFS, or adverse events related with immunotherapy.

Our study also has significant limitations, with inherent variabilities between studies, such as sample size and different statistical methods (HR, CIs) as well as variable modalities of immunotherapies, radiation and chemotherapy doses. Also, in GBM the response to chemo-radiotherapy is significantly affected by the genetic makeup of the tumor (72) and through analysis of biological confounding variables, such as IDH mutations, 1p19q co-deletion, MGMT status etc, could not be done since many trials did not include this information. Importantly, the lack of similarity in treatment modalities and schemes used in these studies limits a truly fair comparison of the possible benefit of immunotherapy. Another limitation in our study is the selection of clinical trials phase II and III, that might bias the results due to the often benefit seen in clinical studies phase II, that do not demonstrate benefits when tested in multiple centers in larger cohorts during phase III trials. Our intention is not to overestimate (or vice versa) the effect of immunotherapy in GBM, but to demonstrate that more clinical trials accounting for dependent and independent-patient variables are needed to truly understand the full potential of immunotherapy in combination with current standard of care.

## Conclusion

In summary, our results demonstrated that the combination of immunotherapy with standard of care chemotherapy and radiation produced no significant survival benefit in patients. Furthermore, the combination was not significantly associated with an increased incidence of grade 3 to 5 SAEs, despite the observed trend. Most of these SAEs were successfully managed clinically, which allow us to conclude that the integration of immunotherapy into the standard of care for GBM is relatively safe. We believe, standardization of clinical trials in regard to immunotherapy and chemo-radiation treatment schemes for GBM treatment is necessary for a more accurate comparison and analysis of these combined treatments and is warranted to fully explore the potential benefits of this therapeutic combination.

## Data Availability Statement

The original contributions presented in the study are included in the article/**Supplementary Material**. Further inquiries can be directed to the corresponding author.

## Author Contributions

ML-V, JS, MD, and HR-G wrote the manuscript. ML-V, KB, HR-G, and JS collected the data. EJL, MP, and RC analyzed the data. All authors contributed to the article and approved the submitted version.

## Funding

This work was supported by the NIH K08NS092895 grant (MD).

## Conflict of Interest

The authors declare that the research was conducted in the absence of any commercial or financial relationships that could be construed as a potential conflict of interest.
