# Supplementary material for: A Comparison Between Chemo-Radiotherapy Combined With Immunotherapy and Chemo-Radiotherapy Alone for the Treatment of Newly Diagnosed Glioblastoma: A Systematic Review and Meta-Analysis
Source: Front Oncol. 2021 May 11;11:662302. doi: 10.3389/fonc.2021.662302 (PMC8144702; doi:10.3389/fonc.2021.662302)
Supplement: Supplementary file 1 [file DataSheet_1.docx]

Supplementary Material

# Supplementary Figures and Tables

**Supplementary Figure 1.** The revised Cochrane risk-of-bias tool for randomized trials.


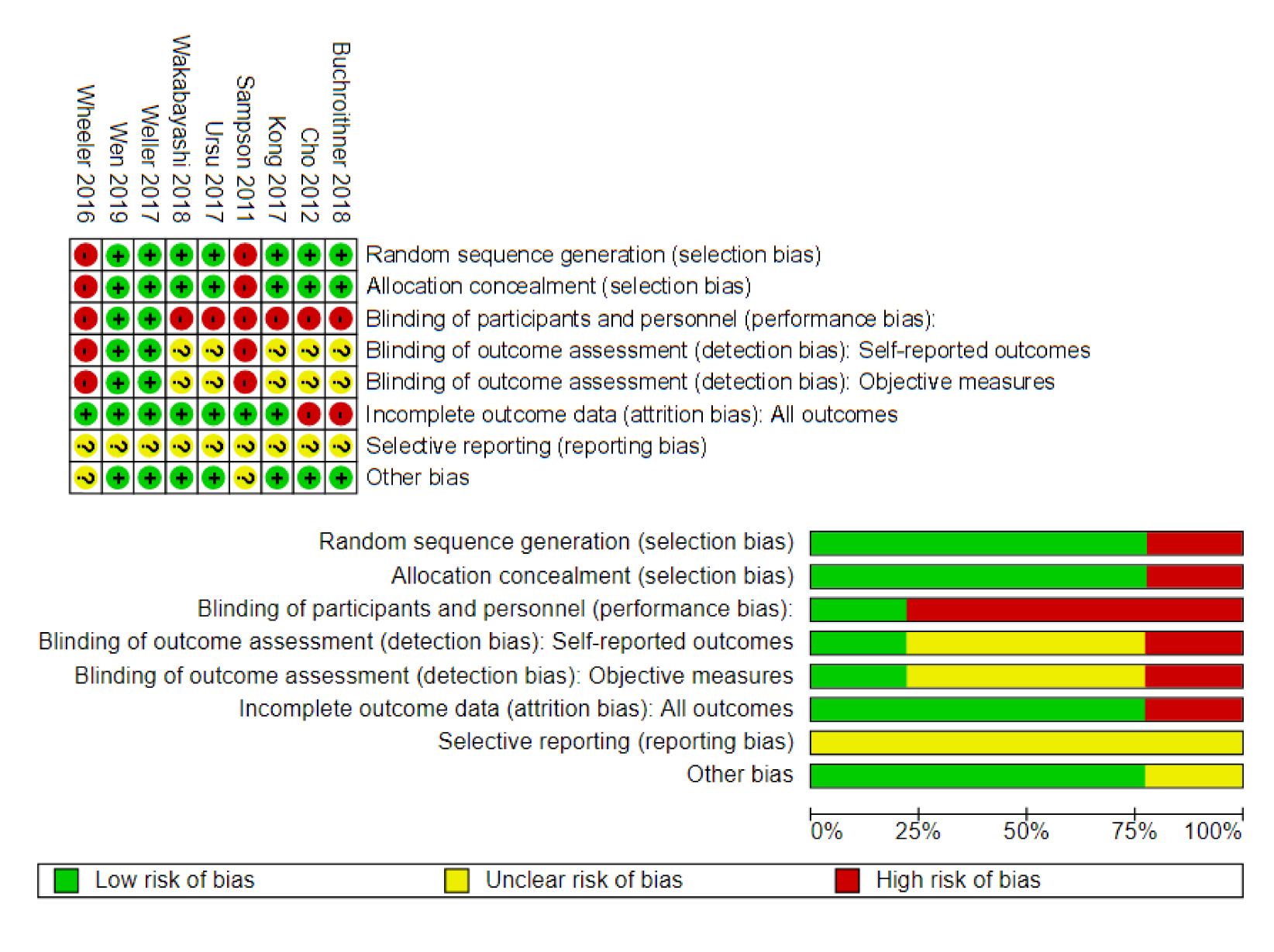


## Supplementary Table 1. Population, Intervention, Control, Outcome, Study Design (PICOS) used in this analysis.

| **Population, Intervention, Control, Outcome, Study Design (PICOS)** | |
| --- | --- |
| **Population** | Patients with newly diagnosed GBM |
| **Intervention** | Chemoradiotherapy and immunotherapy |
| **Control** | Chemoradiotherapy alone |
| **Outcomes** | 1-year OS and PFS  median OS and PFS  SAEs grade 3 to 5 |
| **Study design** | Clinical trials phase II and III |

## Supplementary Table 2. PRISMA checklist.

| **Section/topic** | **#** | **Checklist item** | **Reported on page #** |
| --- | --- | --- | --- |
| **TITLE** | | |  |
| Title | 1 | Identify the report as a systematic review, meta-analysis, or both. | 1 |
| **ABSTRACT** | | |  |
| Structured summary | 2 | Provide a structured summary including, as applicable: background; objectives; data sources; study eligibility criteria, participants, and interventions; study appraisal and synthesis methods; results; limitations; conclusions and implications of key findings; systematic review registration number. | 2 |
| **INTRODUCTION** | | |  |
| Rationale | 3 | Describe the rationale for the review in the context of what is already known. | 3-4 |
| Objectives | 4 | Provide an explicit statement of questions being addressed with reference to participants, interventions, comparisons, outcomes, and study design (PICOS). | 4 Supplementary Table 1 |
| **METHODS** | | |  |
| Protocol and registration | 5 | Indicate if a review protocol exists, if and where it can be accessed (e.g., Web address), and, if available, provide registration information including registration number. | Not performed |
| Eligibility criteria | 6 | Specify study characteristics (e.g., PICOS, length of follow-up) and report characteristics (e.g., years considered, language, publication status) used as criteria for eligibility, giving rationale. | 4-5, Supplementary Table 1 |
| Information sources | 7 | Describe all information sources (e.g., databases with dates of coverage, contact with study authors to identify additional studies) in the search and date last searched. | 5 |
| Search | 8 | Present full electronic search strategy for at least one database, including any limits used, such that it could be repeated. | 5 |
| Study selection | 9 | State the process for selecting studies (i.e., screening, eligibility, included in systematic review, and, if applicable, included in the meta-analysis). | 4-5 |
| Data collection process | 10 | Describe method of data extraction from reports (e.g., piloted forms, independently, in duplicate) and any processes for obtaining and confirming data from investigators. | 5, 6  Figure 1 |
| Data items | 11 | List and define all variables for which data were sought (e.g., PICOS, funding sources) and any assumptions and simplifications made. | 4-5,  Supplementary Text 1,  Supplementary Table 1 |
| Risk of bias in individual studies | 12 | Describe methods used for assessing risk of bias of individual studies (including specification of whether this was done at the study or outcome level), and how this information is to be used in any data synthesis. | 6 |
| Summary measures | 13 | State the principal summary measures (e.g., risk ratio, difference in means). | 2, 6 |
| Synthesis of results | 14 | Describe the methods of handling data and combining results of studies, if done, including measures of consistency (e.g., I^2^) for each meta-analysis. | 6 |

| **Section/topic** | **#** | **Checklist item** | **Reported on page #** |
| --- | --- | --- | --- |
| Risk of bias across studies | 15 | Specify any assessment of risk of bias that may affect the cumulative evidence (e.g., publication bias, selective reporting within studies). | 6, 8-9, Figures 2-7 |
| Additional analyses | 16 | Describe methods of additional analyses (e.g., sensitivity or subgroup analyses, meta-regression), if done, indicating which were pre-specified. | Not performed |
| **RESULTS** | | |  |
| Study selection | 17 | Give numbers of studies screened, assessed for eligibility, and included in the review, with reasons for exclusions at each stage, ideally with a flow diagram. | Figure 1 |
| Study characteristics | 18 | For each study, present characteristics for which data were extracted (e.g., study size, PICOS, follow-up period) and provide the citations. | 4-5, Table 2 and 3, Supplementary Table 1 |
| Risk of bias within studies | 19 | Present data on risk of bias of each study and, if available, any outcome level assessment (see item 12). | 8-9  Figures 2-7 |
| Results of individual studies | 20 | For all outcomes considered (benefits or harms), present, for each study: (a) simple summary data for each intervention group (b) effect estimates and confidence intervals, ideally with a forest plot. | Figures 2-7 |
| Synthesis of results | 21 | Present results of each meta-analysis done, including confidence intervals and measures of consistency. | 8-9 |
| Risk of bias across studies | 22 | Present results of any assessment of risk of bias across studies (see Item 15). | 8-9 |
| Additional analysis | 23 | Give results of additional analyses, if done (e.g., sensitivity or subgroup analyses, meta-regression [see Item 16]). | Not performed |
| **DISCUSSION** | | |  |
| Summary of evidence | 24 | Summarize the main findings including the strength of evidence for each main outcome; consider their relevance to key groups (e.g., healthcare providers, users, and policy makers). | 9 |
| Limitations | 25 | Discuss limitations at study and outcome level (e.g., risk of bias), and at review-level (e.g., incomplete retrieval of identified research, reporting bias). | 18 |
| Conclusions | 26 | Provide a general interpretation of the results in the context of other evidence, and implications for future research. | 9-19 |
| **FUNDING** | | |  |
| Funding | 27 | Describe sources of funding for the systematic review and other support (e.g., supply of data); role of funders for the systematic review. | 19 |

*From:*  Moher D, Liberati A, Tetzlaff J, Altman DG, The PRISMA Group (2009). Preferred Reporting Items for Systematic Reviews and Meta-Analyses: The PRISMA Statement. PLoS Med 6(6): e1000097. doi:10.1371/journal.pmed1000097

## Supplementary Table 3. MOOSE checklist

| **Item No** | **Recommendation** | **Reported on Page No** |
| --- | --- | --- |
| Reporting of background should include | | |
| 1 | Problem definition | 2 |
| 2 | Hypothesis statement | - |
| 3 | Description of study outcome(s) | 5 |
| 4 | Type of exposure or intervention used | 4 |
| 5 | Type of study designs used | 2 |
| 6 | Study population | 4,5 |
| Reporting of search strategy should include | | |
| 7 | Qualifications of searchers (eg, librarians and investigators) | 6 |
| 8 | Search strategy, including time period included in the synthesis and key words | 5 |
| 9 | Effort to include all available studies, including contact with authors | 5 |
| 10 | Databases and registries searched | 5 |
| 11 | Search software used, name and version, including special features used (eg, explosion) | - |
| 12 | Use of hand searching (eg, reference lists of obtained articles) | 5 and figure 1 |
| 13 | List of citations located and those excluded, including justification | 5, table 1 |
| 14 | Method of addressing articles published in languages other than English | Table 1 |
| 15 | Method of handling abstracts and unpublished studies | - |
| 16 | Description of any contact with authors | - |
| Reporting of methods should include | | |
| 17 | Description of relevance or appropriateness of studies assembled for assessing the hypothesis to be tested | 5 |
| 18 | Rationale for the selection and coding of data (eg, sound clinical principles or convenience) | - |
| 19 | Documentation of how data were classified and coded (eg, multiple raters, blinding and interrater reliability) | - |
| 20 | Assessment of confounding (eg, comparability of cases and controls in studies where appropriate) | - |
| 21 | Assessment of study quality, including blinding of quality assessors, stratification or regression on possible predictors of study results | 6 |
| 22 | Assessment of heterogeneity | 6 |
| 23 | Description of statistical methods (eg, complete description of fixed or random effects models, justification of whether the chosen models account for predictors of study results, dose-response models, or cumulative meta-analysis) in sufficient detail to be replicated | 6 |
| 24 | Provision of appropriate tables and graphics | 24,27 supplementary files |
| Reporting of results should include | | |
| 25 | Graphic summarizing individual study estimates and overall estimate | - |
| 26 | Table giving descriptive information for each study included | Table 3 |
| 27 | Results of sensitivity testing (eg, subgroup analysis) | - |
| 28 | Indication of statistical uncertainty of findings | 8-10 |

| **Item No** | **Recommendation** | **Reported on Page No** |
| --- | --- | --- |
| Reporting of discussion should include | | |
| 29 | Quantitative assessment of bias (eg, publication bias) | 6 |
| 30 | Justification for exclusion (eg, exclusion of non-English language citations) | 5, table 1 |
| 31 | Assessment of quality of included studies | 7, table 2 |
| Reporting of conclusions should include | | |
| 32 | Consideration of alternative explanations for observed results | 9-18 |
| 33 | Generalization of the conclusions (ie, appropriate for the data presented and within the domain of the literature review) | 18,19 |
| 34 | Guidelines for future research | 18,19 |
| 35 | Disclosure of funding source | 19 |

*From*: Stroup DF, Berlin JA, Morton SC, et al, for the Meta-analysis Of Observational Studies in Epidemiology (MOOSE) Group. Meta-analysis of Observational Studies in Epidemiology. A Proposal for Reporting. *JAMA*. 2000;283(15):2008-2012. doi: 10.1001/jama.283.15.2008.

**Supplementary Table 4:** GRADE ratings and their interpretation Symbol Quality Interpretation

| Symbol | Quality | Interpretation |
| --- | --- | --- |
| ⊕⊕⊕⊕ | High | We are very confident that the true effect lies close to that of the estimate of the effect. |
| ⊕⊕⊕ | Moderate | We are moderately confident in the effect estimate: the true effect is likely to be close to the estimate of the effect, but there is a possibility that is substantially different. |
| ⊕⊕ | Low | Our confidence in the effect estimate is limited: the true effect may be substantially different from the estimate or the effect. |
| ⊕ | Very Low | We have little confidence in the effect estimate: the true effect is likely to be substantially different from the estimate of effect. |

**Table taken from the GRADE Handbook, available at: <http://gdt.guidelinedevelopment.org/app/handbook/handbook.html#h.9rdbelsnu4iy>

| Quality assessment | | | | | | | No of patients included | | Effect | Quality | Importance |
| --- | --- | --- | --- | --- | --- | --- | --- | --- | --- | --- | --- |
| No of studies | **Design** | **Risk of bias** | **Inconsistency** | **Indirectness** | **Imprecision** | **Other considerations** | **Standard of care** | **Standard of care plus immunotherapy** | **P value** |  |  |
| 1 -year overall survival | | | | | | | | | | | |
| 9 | RCT _a_  Non-RCT _a_ | Serious _b, c_ | Not serious _e_ | Serious _c, d_ | Not serious _e_ | none | 656(52.9 %)/  1239 | 583 (47%)/ 1239 | 0.15 | ⊕⊕⊕ Moderate | Critical |
| 1 -year progression free survival | | | | | | | | | | | |
| 7 | RCT _a, f, g_ | Serious _b, c_ | Not serious _e_ | Serious _c, d_ | Not serious _e_ | none | 480 (49.1%) /976 _f, g_ | 496 (50.8%)/976 _f, g_ | 0.17 | ⊕⊕⊕ Moderate | Critical |
| Severe adverse events (grade 3 to 5) | | | | | | | | | | | |
| 4 | RCT _a, h_ | Serious _b, c_ | Not serious _e_ | Serious _c_ | Serious _e, h_ | none | 542 (48.8 %)/1110 _h_ | 568 (51.1%)/1110 _h_ | 0.81 | ⊕⊕ Low | Critical |

**Supplementary Table 5 for assessing the GRADE criteria**

**Question:** Does standard of care plus immunotherapy improves 1-year overall survival, 1-year progression free survival and decreases severe adverse events (grade 3 to 5) in comparison with standard of care alone, for newly diagnosed GBM?

**Abbreviations:**  RCT = randomized controlled trial, Non-RCT = non- randomized controlled trial.

1. 7 trials were randomized (Buchroithner, Cho, Kong, Ursu, Wakabayashi, Weller and Wen), 1 trial was not randomized (Sampson) and another trial used a non-adequate method of randomization (Wheeler ‘s).
2. Risk of bias related to blinding of participants and personnel was present. Only two studies were double blinded (Weller and Wen’s), Ursu’s was single blinded and the rest were open-label or did not specify. This may cause overestimation of efficacy or underestimation of the risk of serious adverse events; therefore, the risk of bias was rated as serious. In addition, not all the studies adequately described severe adverse events grade 3 or higher.
3. Concern of indirectness was noted due to variability in several aspects among studies. The number of people included and analyzed in each trial varied widely, the route of administration was different between trials. Also, there was variability in the follow up period, and the timing of when immunotherapy was initiated. There was significant variation among concomitant and maintenance therapy regiments too. The targeted dose, as well as the number of doses administered (length of treatment) were different. In addition, there was significant heterogeneity in the SAE reporting criteria used by each of the clinical trials included in this meta-analysis. These factors could potentially affect clinical outcomes and toxicity rate.
4. The selection criteria and overall health status of the patients enrolled heavily weighted on the overall outcome of the patients in clinical trials. Sampson et al trial included patients with KPS score of 100, Weller et al trial included patients with RPA class IV or higher, which may influence the overall outcome. There was also indirect comparison by using a historical control group instead of a true control arm, like in Sampson’s trial.
5. The sample size varied among studies: Buchroithner 76 patients, Cho 34 patients, Wen 124 patients, Kong 180 patients, Sampson 35 patients, Weller 405 patients, Ursu 81 patients, Wakabayashi 122 patients and Wheeler 182 patients, thus weighing differently on the overall analysis.
6. 2 studies did not describe 1-year progression free survival data (PFS). Ursu did not include information about 1-year PFS, whereas Wheeler did not include information of 1-year PFS specifically for the GBM subgroup.
7. Two Phase II studies that did not described 1-year PFS analysis appropriately were not included in the GRADE analysis.
8. Only four studies included an adequate description of SAEs grade 3 to 5 (Wen, Buchroithner, Kong and Weller). Only these studies were included in the GRADE analysis.

**Supplementary Table 6.** Administration routes of immunotherapy

| **First author** | **Routes of administration** |
| --- | --- |
| Buchroithner | intravenous |
| Cho | intravenous |
| Kong | intravenous |
| Sampson | intradermal |
| Ursu | intracranial |
| Wakabayashi | intravenous |
| Weller | intradermal |
| Wen | intradermal |
| Wheeler | intracranial, intravenous/oral |

**Supplementary Table 7.** Pool data showing median and 1-year OS and PFS in the clinical trials included.

|  | **Median OS (months)** | | **1-year Survival (%)** | |
| --- | --- | --- | --- | --- |
| **Author** | **Chemoradiation Alone** | **Chemoradiation + Immunotherapy** | **Chemoradiation Alone** | **Chemoradiation + Immunotherapy** |
| Buchroithner | 18.6 | 18.5 | 64.52 | 76.88 |
| Cho | 15 | 31.9 | 75 | 88.9 |
| Kong | 16.9 | 22.5 | 75.2 | 78.2 |
| Sampson | 15 | 23.6 | 70.6 | 100 |
| Ursu | 18 | 17 | 81.01 | 69.36 |
| Wakabayashi | 20.3 | 24 | 76.18 | 88.39 |
| Weller | 20 | 20.1 | 82 | 78 |
| Wen | 15 | 17 | 67.3 | 71.45 |
| Wheeler | 13.7 | 16.7 | 57.8 | 62.8 |
| **Median** | **16.9** | **20.1** | **75** | **78** |
|  | **Median PFS (months)** | | **1-year PFS (%)** | |
| **Author** | **Chemoradiation Alone** | **Chemoradiation + Immunotherapy** | **Chemoradiation Alone** | **Chemoradiation + Immunotherapy** |
| Buchroithner | 6.9 | 6.7 | 28.4 | 24.5 |
| Cho | 8 | 8.5 | 31.13 | 51.18 |
| Kong | 5.4 | 8.1 | 22.6 | 28.3 |
| Sampson | 6.3 | 15.2 | 23.5 | 63.6 |
| Ursu | 9 | 9 | NA | NA |
| Wakabayashi | 10.1 | 8.5 | 38.01 | 35.54 |
| Weller | 7.4 | 8 | 33 | 32 |
| Wen | 9 | 11.2 | 31.38 | 43.81 |
| Wheeler | NA | NA | NA | NA |
| **Median** | **7.7** | **8.5** | **31.3** | **35.5** |

**Supplementary Table 8.** Pool data showing SAEs in the clinical trials included.

| **Author** | **Chemoradiation alone (n) SAEs** | **Chemoradiation alone (n) total included for safety analysis** | **Chemoradiation alone SAEs (%)** | **Chemoradiation + Immunotherapy (n)** | **Chemoradiation +Immunotherapy (n) total included for safety analysis** | **Chemoradiation +Immunotherapy SAEs (%)** |
| --- | --- | --- | --- | --- | --- | --- |
| Buchroithner | 12 | 42 | 28.57142857 | 18 | 34 | 52.94117647 |
| Cho | NA | NA | NA | NA | NA | NA |
| Kong | 32 | 85 | 37.64705882 | 49 | 85 | 57.64705882 |
| Sampson | NA | NA | NA | NA | NA | NA |
| Ursu | NA | NA | NA | NA | NA | NA |
| Wakabayashi | NA | NA | NA | NA | NA | NA |
| Weller | 105 | 372 | 28.22580645 | 87 | 369 | 23.57723577 |
| Wen | 35 | 43 | 81.39534884 | 47 | 80 | 58.75 |
| Wheeler | NA | NA | NA | NA | NA | NA |
| **Total** | **184** | **542** |  | **201** | **568** |  |
| **Median** |  |  | **33.1** |  |  | **55.2** |

**Supplementary Table 9.** Criteria for the most common SAEs grade 3 to 5 in the included clinical trials (with some variations according to the version used).

|  | **Common Terminology Criteria for Adverse Events v3.0 (CTCAE)** | | |
| --- | --- | --- | --- |
| **Symptom** | **Grade 3** | **Grade 4** | **Grade 5** |
| headache | Severe pain; pain or analgesics severely interfering with ADL | Disabling | ---- |
| nausea | Inadequate oral caloric or fluid intake; IV fluids, tube feedings, or TPN indicated ≥24 hrs | Life-threatening consequences | Death |
| vomiting | ≥6 episodes in 24 hrs; IV fluids, or TPN indicated ≥24 hrs | Life-threatening consequences | Death |
| seizures | Seizures in which consciousness is altered; poorly controlled seizure disorder, with breakthrough generalized seizures despite medical intervention | Seizures of any kind which are prolonged, repetitive, or difficult to control (e.g., status epilepticus, intractable epilepsy) | Death |
| constipation | Symptoms interfering with ADL; obstipation with manual evacuation indicated | Life-threatening consequences (e.g., obstruction, toxic megacolon) | Death |
| diarrhea | Increase of ≥7 stools per day over baseline; incontinence; IV fluids ≥24 hrs; hospitalization; severe increase in ostomy output compared to baseline; interfering with ADL | Life-threatening consequences (e.g., hemodynamic collapse) | Death |
| weakness | Symptomatic and interfering with ADL | Life-threatening; disabling | Death |
| anorexia | Associated with significant weight loss or malnutrition (e.g., inadequate oral caloric and/or fluid intake); IV fluids, tube feedings or TPN indicated | Life-threatening consequences | Death |
| pyrexia | >40.0°C (>104.0°F) for ≤24 hrs | >40.0°C (>104.0°F) for >24 hrs | Death |
| increase transaminases | ALT/AST: >5.0 – 20.0 x ULN | ALT/AST: >20.0 x ULN | ---- |
| increase lipases | >2.0 – 5.0 x ULN | >5.0 x ULN | ---- |
| increase intracranial pressure | Severe symptoms or neurological deficit interfering with ADL | Disabling | Death |
| rash/allergic reactions | Symptomatic bronchospasm, with or without urticaria; parenteral medication(s) indicated; allergy-related edema/angioedema; hypotension | Anaphylaxis | Death |
| lymphopenia | <500-200 mm^3^, <0.5-0.2x10^9^/L | <200/mm^3^, <0.2X10^9^/L | Death |
| thrombocytopenia | <50,000-25,000/mm3, <50-25x10^9^/L | <25,000/mm^3^, <25x10^9^/L | Death |
| neutropenia | <1000-500/mm^3^  <1.0-0.5 X10^9^/L | <500/mm^3^  <0.5x10^9^/L | Death |

ADL: Activities of daily living
